# Exogenous Calcium Delays Grape Berry Maturation in the White cv. Loureiro While Increasing Fruit Firmness and Flavonol Content

**DOI:** 10.3389/fpls.2021.742887

**Published:** 2021-08-27

**Authors:** Viviana Martins, Marianne Unlubayir, António Teixeira, Arnaud Lanoue, Hernâni Gerós

**Affiliations:** ^1^Department of Biology, Centre of Molecular and Environmental Biology, University of Minho – Campus de Gualtar, Braga, Portugal; ^2^Centre for the Research and Technology of Agro-Environmental and Biological Sciences, University of Trás-os-Montes and Alto Douro, Vila Real, Portugal; ^3^EA 2106 Biomolécules et Biotechnologie Végétales, UFR des Sciences Pharmaceutiques, Université de Tours, Tours, France; ^4^Department of Biological Engineering, Centre of Biological Engineering (CEB), University of Minho – Campus de Gualtar, Braga, Portugal

**Keywords:** anthocyanins, amino acids, calcium, flavonols, fruit firmness, grape berry ripening, polyphenolic metabolism, white grape cultivars

## Abstract

Vineyard calcium (Ca) sprays have been increasingly used by grape growers to improve fruit firmness and thus maintain quality, particularly in periods of heavy rains and hail. The observation that Ca visibly modified berry size, texture, and color in the most prominent white cultivar of the DOC region ‘Vinhos Verdes’, cultivar (cv.) Loureiro, led us to hypothesize that Ca induced metabolic rearrangements that resulted in a substantial delay in fruit maturation. Targeted metabolomics by ultra-performance liquid chromatography coupled to mass spectrometry and directed transcriptomics were thus combined to characterize the metabolic and transcriptional profiles of cv. Loureiro berries that, together with firmness, °Brix, and fruit weight measurements, allowed to obtain an integrated picture of the biochemical and structural effects of Ca in this cultivar. Results showed that exogenous Ca decreased amino acid levels in ripe berries while upregulating *PAL1* expression, and stimulated the accumulation of caftaric, coutaric, and fertaric acids. An increase in the levels of specific stilbenoids, namely *E*-piceid and *E*-ω-viniferin, was observed, which correlated with the upregulation of *STS* expression. Trace amounts of anthocyanins were detected in berries of this white cultivar, but Ca treatment further inhibited their accumulation. The increased berry flavonol content upon Ca treatment confirmed that Ca delays the maturation process, which was further supported by an increase in fruit firmness and decrease in weight and °Brix at harvest. This newly reported effect may be specific to white cultivars, a topic that deserves further investigation.

## Introduction

Calcium (Ca) supplements have been increasingly used in fresh fruits and vegetables toward improved fitness, sanitation, nutritional enrichment, and decay prevention (Martín-Diana et al., 2007). Diverse supplementation strategies have been optimized, from routinely spraying the fruits throughout their development in the tree, applying a single treatment at pre-harvest, or supplying Ca only at postharvest, through spraying, dipping, or even impregnation techniques (Saftner et al., 1997; Martín-Diana et al., 2005, 2007; Manganaris et al., 2007; Wang et al., 2014; Correia et al., 2019). The refinement of these methodologies showed that the beneficial effects of Ca can be achieved at both pre-harvest and postharvest stages, supporting its potential to protect the grape berries against abiotic and biotic stresses (Alcaraz-López et al., 2005; Romanazzi et al., 2012; Ciccarese et al., 2013; González-Fontes et al., 2017; Aldon et al., 2018). Accordingly, increased fruit resistance to infection by *Botrytis cinerea* during storage was achieved by Ca dips after harvest (Fu et al., 2020), an effect that was also reported upon vineyard Ca sprays between fruit set and veraison stages (Amiri et al., 2009; Ciccarese et al., 2013). In line with these observations, Ca sprays during berry development reduce the incidence of microcracks on the fruit surface and the lodging of filamentous fungi in these structures, reducing fruit decay at postharvest (Martins et al., 2020b, 2021a).

The effects of Ca on fruit texture are connected to its structural function in the cell wall and membranes, mediating cross-links between pectin molecules, and inhibiting the activity of polygalacturonases responsible for fruit softening (Hocking et al., 2016; Martins et al., 2020b), but a myriad of developmental and stress–response processes mediated by Ca may occur because it is a pivotal secondary messenger (González-Fontes et al., 2017; Aldon et al., 2018). Recent studies reported increased bulk anthocyanin content in berries cultivar (cv.) Manicure Finger sprayed with Ca around veraison stage (Yu et al., 2020). In contrast, a general repression in the synthesis of anthocyanins in the grape berry was shown through a targeted metabolomics approach, following Ca treatments throughout the entire fruiting season in vineyards cv. Vinhão (Martins et al., 2020a). This effect was also reported in grape cell cultures cv. Gamay Fréaux var. Teinturier, which became significantly less pigmented upon Ca treatment, due to a general repression of the entire flavonoid pathway (Martins et al., 2018, 2020a). These effects were underlaid by the Ca-driven regulation of core enzymes of secondary metabolism, at gene expression and protein activity levels, besides vacuolar transporters mediating anthocyanin accumulation (Martins et al., 2018). Transcriptomics studies in berries cv. Manicure Finger also showed the involvement of Ca-activated transcription factors in the regulation of anthocyanin levels (Yu et al., 2020). In cv. Vinhão berries, the inhibition of anthocyanin synthesis was accompanied by a general accumulation of stilbenoids, including *E*-resveratrol, *E-ε*-viniferin, *E*-piceid, and pallidol, demonstrating the powerful ability of Ca in diverting polyphenolic biosynthetic routes (Martins et al., 2020a).

The studies reported above provided a good overview of the effects of Ca over the metabolism of red wine grape cultivars (Martins et al., 2018, 2020a; Yu et al., 2020); however, how the phenolics metabolism of berries from white cultivars is affected by Ca is still puzzling. Previous studies indicated a qualitative improvement in the general color of grape berries of the white cv. Asgari (Amiri et al., 2009), but information on the metabolic mechanisms underlying these effects is lacking. The present study aimed at filling a gap in the literature, following the observation that Ca visibly modified berry size, texture, and color in the white cv. Loureiro, in two consecutive seasons. Our main hypothesis is that Ca induces specific metabolic rearrangements that result in a substantial delay in fruit maturation. Integrated metabolomics and directed transcriptomics were combined to study the effect of Ca over key genes involved in polyphenol biosynthesis and in grape berry metabolic profile at harvest time. The determination of technical and biochemical parameters such as firmness, °Brix, and fruit weight further enlightened the mechanisms of Ca-driven modulation of fruit structure and metabolism in cv. Loureiro, possibly posing as a model for other white cultivars.

## Materials and Methods

### Vineyard Treatments and Sample Collection

Field trials were performed in grapevines cv. Loureiro, the most prominent white cultivar of the Portuguese DOC region of ‘Vinhos Verdes’ (edaphoclimatic conditions specified in Supplementary Figure 1), cultivated in a commercial vineyard with coordinates: N41°28′28″ latitude, W8°34′59″ longitude, 165 m altitude. The plants were oriented in southwest to northeast, spaced at 2.2 m between rows, 1.0 m along the row and trained on a vertical shoot position trellis system, uniformly pruned on a unilateral Royat cordon. Grapevine aerial parts were evenly sprayed with a solution of 2% (w/v) CaCl_2_ and 0.1% (v/v) Silwet L-77 used as a surfactant, as previously optimized (Saftner et al., 1997; Martins et al., 2020a, b, 2021a). Treatments were performed in the early morning, and 3 L of the solution was used for every 10 plants. Three applications were performed throughout the fruiting season, every 30 days, the first performed at the pea size (E-L 31; Coombe, 1995), the second performed at veraison stage (E-L 35), and the last performed 1 week before harvest (E-L 38). Control plants were sprayed with a solution containing the surfactant agent only. Both control and Ca-treated grapevines were healthy, cultivated under the same microclimate, and subjected to the same routine phytosanitary treatments with Topaze and Ridomil Gold R WG, according to the instructions of the suppliers. Rows of control and Ca-treated vines were intercalated with vines with no treatment At the harvest time, berries were randomly collected from 10 Ca-treated and control grapevines. Six independent sets (*n* = 6) of approximately 30 berries each were frozen immediately in liquid nitrogen and stored at −80°C for further characterization of metabolite profile. The determination of technical/biochemical parameters and gene expression analysis were performed in three pools of two independent sets (*n* = 3).

### Determination of Berry Weight, Ca Content, °Brix, and Firmness

Grape berry fresh weight was assessed with an analytical scale Mettler Toledo AG245 (Martins et al., 2020a). Ca content was determined in profusely washed berries, using a previously optimized adaptation of the technique described by Spare (1964). Briefly, berries were ground in liquid nitrogen and 200 mg of fresh weight were used for extraction of soluble contents in 1.5 mL milli-Q H_2_O. After centrifuging the extracts at 12,000 × *g* for 3 min, 300 μL of 0.008% (w/v) murexide reagent was added to 500 μl of the supernatant. Following incubation for 15 min at RT, the OD_490nm_ was recorded and Ca concentration was determined through a calibration curve of CaCl_2_ solution at 2–150 μM (Martins et al., 2020b). The °Brix was determined in aliquots of grape juice using a digital wine refractometer Hanna HI 96813, as described previously (Martins et al., 2020c). Fruit firmness was assessed in 24 intact fruits containing the pedicel, by determining the tension necessary to perforate the fruit skin. Tests were performed on a Shimadzu (model AG-IS) equipped with a 50-N load cell and a 1-mm diameter needle. Force–stroke plots were assembled in Trapezium 2.0 Software and results were expressed in MPa, as described previously (Martins et al., 2020b).

### Characterization of the Grape Berry Metabolic Profile

Grape berries were ground in liquid nitrogen and converted into a fine powder. Metabolites were extracted from freeze-dried samples, using a proportion of 1 mL of 80% (v/v) methanol per 25 mg of dry weight. Samples were sonicated for 30 min and macerated overnight at 4°C in the dark, centrifuged at 18,000*g* for 10 min, and the supernatants were recovered. Ultra-performance liquid chromatography coupled to mass spectrometry (UPLC–MS)-targeted metabolomic analysis was performed as optimized previously (Billet et al., 2018a,b,c; Martins et al., 2020a), using an ACQUITY UPLC system coupled to a photo diode array detector and a Xevo TQD mass spectrometer (Waters, Milford, MA, United States) equipped with an electrospray ionization source controlled by Masslynx 4.1 software (Waters, Milford, MA, United States). Analyte separation was achieved by using a Waters Acquity HSS T3 C18 column (150 × 2.1 mm, 1.8 μm) with a flow rate of 0.4 mL/min at 55°C. Chromatographic separation and identification of analytes were achieved as optimized previously, using the same standards specified by Martins et al. (2020a, 2021b). UPLC–MS analyses were achieved using the selected ion monitoring (SIM) mode of the targeted molecular ions. SIM chromatograms were integrated using the subroutine QuanLynx 4.1 for data mining. Peak integration was performed using the ApexTrack algorithm with a mass window of 0.1 Da and relative retention time window of 1 min followed by Savitzky–Golay smoothing (iteration = 1 and width = 1). To evaluate the robustness of measurements and analytical variability, a pool of all samples was prepared to obtain a quality control sample and the samples were randomly injected. Relative quantification was determined for L-proline (m1), L-leucine (m2), L-isoleucine (m3), L-phenylalanine (m4), L-tyrosine (m5), L-tryptophan (m6), cyanidin-3-*O*-glucoside (m7), peonidin-3-*O*-glucoside (m8), delphinidin-3-*O*-glucoside (m9), cyanidin-3-*O*-(6-*O*-acetyl)-glucoside (m10), malvidin-3-*O*-glucoside (m11), malvidin-3-*O*-(6-*O*-acetyl)-glucoside (m12), petunidin-3-*O*-(6-*p*-coumaroyl)-glucoside (m13), malvidin-3-*O*-(6-*p*-coumaroyl)-glucoside (m14), malvidin-3,5-*O*-diglucoside (m15), gallic acid (m16), citric acid (m17), *E*-resveratrol (m18), *E*-piceatannol (m19), catechin (m20), epicatechin (m21), coutaric acid (m22), caftaric acid (m23), fertaric acid (m24), *E*-piceid (m25), kaempferol-3-*O*-glucoside (m26), pallidol (m27), *E*-ε-viniferin (m28), *E*-ω-viniferin (m29), *E*-δ-viniferin (m30), quercetin-3-*O*-glucoside (m31), quercetin-3-*O*-glucuronide (m32), myricetin-hexoside 1 (m33), myricetin-hexoside 2 (m34), quercetin derivative (m35), procyanidin B1 (m36), procyanidin B2 (m37), procyanidin B3 (m38), procyanidin B4 (m39), kaempferol-3-*O*-rutinoside (40), procyanidin gallate (41), procyanidin trimer 1 (42), and procyanidin trimer 2 (43).

### RNA Extraction and Quantitative Real-Time PCR Analysis

Total RNA was extracted from 0.3 g of freshly ground samples according to the method of Reid et al. (2006), as previously optimized (Martins et al., 2020a, b). RNA was purified with the GRS Total RNA kit – Plant (Grisp Research Solutions, Porto, Portugal), and the cDNA was obtained from 1 μg of mRNA by reverse transcription with an Xpert cDNA Synthesis Kit and oligo (dT) primers (Grisp Research Solutions). Quantitative real-time PCR (qRT-PCR) reactions were performed in triplicate, as previously described (Martins et al., 2020a, b). The sequences of the gene-specific primers used are detailed in Supplementary Table 1. Genes encoding core enzymes of secondary metabolism were selected, namely, *PAL1* (phenylalanine ammonia lyase), *STS* (stilbene synthase), *CHS3* (chalcone synthase), *CHI1* (chalcone isomerase), *F3′5′H* (flavonoid 3′,5′-hydroxylase), *F3H1* (flavanone 3-hydroxylase), *FLS1* (flavonol synthase), *DFR* (dihydroflavonol 4-reductase), *LAR1* (leucoanthocyanidin reductase), *ANS* (anthocyanidin synthase), *BAN* and *ANR* (anthocyanidin reductases), and UDP–glucose:flavonoid-3-O-glucosyltransferase (*UFGT*) (Jeong et al., 2004; Bogs et al., 2005; Castellarin et al., 2007; Tavares et al., 2013; Martins et al., 2018). The expression of *LAC* encoding a laccase involved in the oxidation of *E*-resveratrol was also studied, together with key genes involved in cell wall and cuticle structure, namely, *PME1* (pectin methylesterase), *PG1* (polygalacturonase), *EXP6* (expansin), *CesA3* (cellulose synthase), *CER9* (E3 ubiquitin ligase), and *CYP15* (cytochrome P450 monooxygenase/hydroxylase) (Martins et al., 2018, 2020b). Dissociation curves allowed confirmation of the specificity of the PCR reactions. Expression of target genes was normalized to that of the reference genes glyceraldehyde 3-phosphate dehydrogenase (*GAPDH*) and actin (*ACT1*) (Martins et al., 2020a) using the ΔΔCq method in CFX Manager Software 3.1 (Bio-Rad Laboratories, Inc., Hercules, CA, United States).

### General Statistical Analysis

Results were statistically analyzed through the Student’s *t*-test in Prism6 (GraphPad Software, Inc.). The significance level of differences between the control and the Ca-treated samples is marked in graphs with asterisks: ^∗^*P* ≤ 0.05; ^∗∗^*P* ≤ 0.01; ^∗∗∗^*P* ≤ 0.001; ^****^P ≤ 0.0001. A multivariate statistical data analysis (MVA) of the samples was performed with SIMCA P+ version 15 (Umetrics AB, Umeå, Sweden), after mean-centering all variables and scaling unit-variance. Metabolic variables affected by Ca treatment were revealed through the principal component analysis (PCA) applied as the unsupervised MVA method.

## Results

**FIGURE 1:**
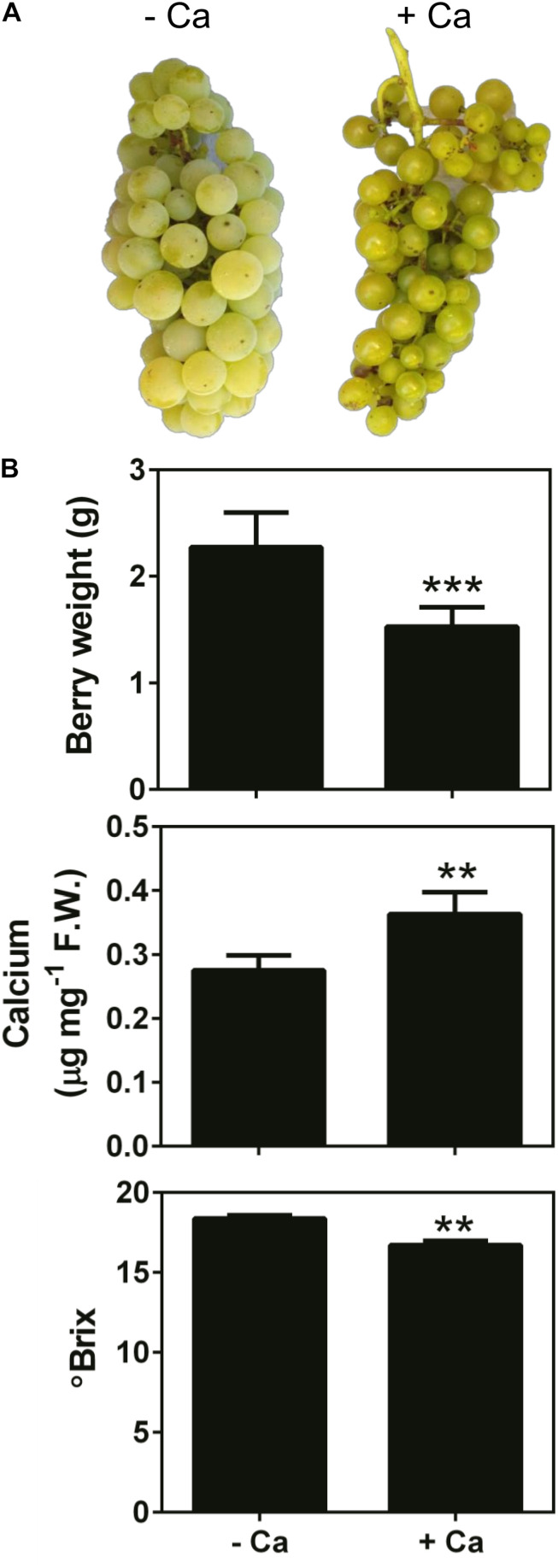
Appearance **(A)**, weight, Ca content, and °Brix **(B)** of mature berries from vines cv. Loureiro treated with Ca (+ Ca) or without treatment (– Ca). Images in panel **(A)** are representative of two consecutive seasons. Results are expressed as mean ± SD and asterisks denote statistical significance as compared to control (– Ca): **P ≤ 0.01; ***P ≤ 0.001; *n* = 3.

**FIGURE 2:**
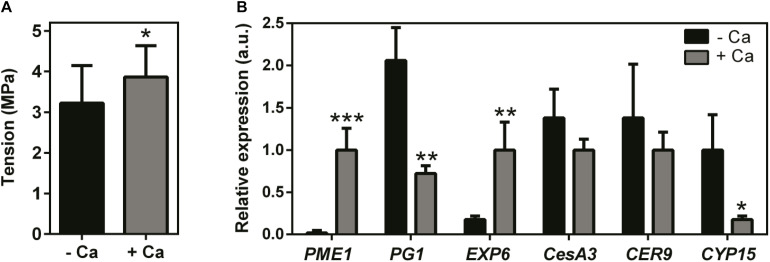
Firmness **(A)** and transcript levels of genes involved in cell wall and cuticle structures **(B)**, in berries from vines cv. Loureiro treated with Ca (+ Ca) or without treatment (– Ca). Firmness is expressed as the tension necessary to perforate the fruit skin; *n* = 24. Transcript levels are shown for pectin methylesterase (*PME1*), polygalacturonase (*PG1*), expansin (*EXP6*), cellulose synthase (*CesA3*), E3 ubiquitin ligase (*CER9*), and cytochrome P450 monooxygenase/hydroxylase (*CYP15*). Results are expressed as mean ± SD and asterisks denote statistical significance as compared to control (– Ca): *P ≤ 0.05; **P ≤ 0.01; ***P ≤ 0.001; *n* = 3.

Results showed that Ca treatment throughout the fruiting season visibly modified the size (decreased), texture, and color of the berries at harvest time, and these effects were consistent in two consecutive seasons (Figure 1A). Accordingly, the fresh weight of berries from Ca-treated plants was 35% lower than that of the control fruits. In parallel, Ca sprays increased fruit Ca content by 30% and decreased the °Brix from 18.4 to 16.7 °B, in line with the immature appearance of the fruits (Figure 1B). In addition, the force necessary to perforate the skin of berries from Ca-treated plants, expressed as tension, was significantly higher than that of the control fruits (Figure 2A). This change was associated to a 56-fold increase in the expression of cell wall *PME1* (Figure 2B). Likewise, transcript levels of *EXP6* also increased by 5.6-fold. In contrast, the expression of *PG1* decreased by 66% upon Ca treatment. The same effect was observed for *CYP15* involved in the cuticle structure, that was downregulated by 82%. Other genes involved in the cell wall and cuticle structures, namely *CesA3* and *CER9* were not significantly affected by the Ca treatment.

**FIGURE 3:**
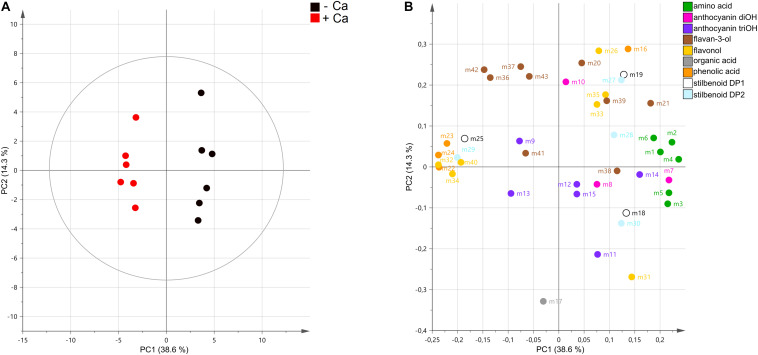
Unsupervised classification using the principal component analysis on metabolomic data from grape berries from vines cv. Loureiro treated with Ca (+ Ca) or without treatment (– Ca) (*n* = 6). Variables in the score plot **(A)** were colored according to the treatment, and variables in loading plot **(B)** were colored according to the metabolic class. Numbers indicate the ID of metabolites, as follows: L-proline (m1), L-leucine (m2), L-isoleucine (m3), L-phenylalanine (m4), L-tyrosine (m5), L-tryptophan (m6), cyanidin-3-*O*-glucoside (m7), peonidin-3-*O*-glucoside (m8), delphinidin-3-*O*-glucoside (m9), cyanidin-3-*O*-(6-*O*-acetyl)-glucoside (m10), malvidin-3-*O*-glucoside (m11), malvidin-3-*O*-(6-*O*-acetyl)-glucoside (m12), petunidin-3-*O*-(6-*p*-coumaroyl)-glucoside (m13), malvidin-3-*O*-(6-*p*-coumaroyl)-glucoside (m14), malvidin-3,5-*O*-diglucoside (m15), gallic acid (m16), citric acid (m17), *E*-resveratrol (m18), *E*-piceatannol (m19), catechin (m20), epicatechin (m21), coutaric acid (m22), caftaric acid (m23), fertaric acid (m24), *E*-piceid (m25), kaempferol-3-*O*-glucoside (m26), pallidol (m27), *E*-ε-viniferin (m28), *E*-ω-viniferin (m29), *E*-δ-viniferin (m30), quercetin-3-*O*-glucoside (m31), quercetin-3-*O*-glucuronide (m32), myricetin-hexoside 1 (m33), myricetin-hexoside 2 (m34), quercetin derivative (m35), procyanidin B1 (m36), procyanidin B2 (m37), procyanidin B3 (m38), procyanidin B4 (m39), kaempferol-3-*O*-rutinoside (40), procyanidin gallate (41), procyanidin trimer 1 (42), and procyanidin trimer 2 (43).

**FIGURE 4:**
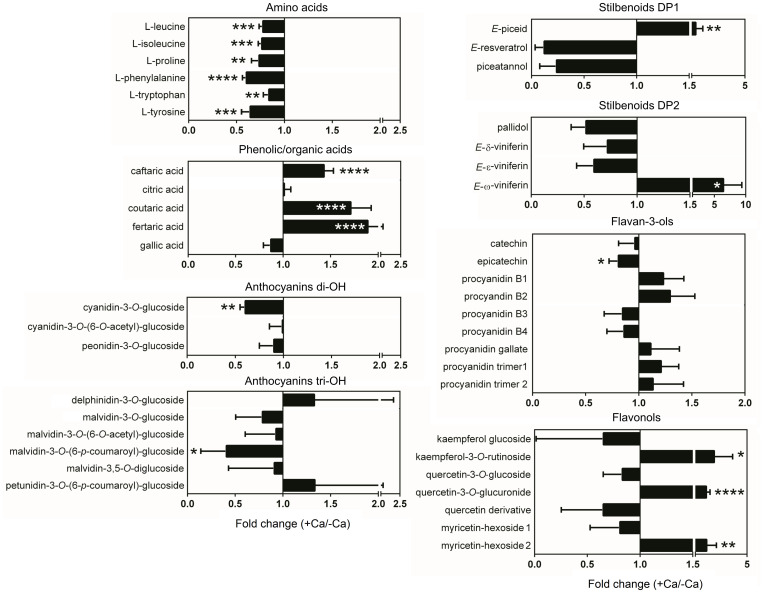
Effect of Ca on the metabolite profile of grape berries cv. Loureiro. Results are expressed as fold changes of the mean ± SD values obtained for Ca treatment (+ Ca) relative to the control (– Ca), and asterisks indicate statistical significance of + Ca *vs.* – Ca for each metabolite: *P ≤ 0.05; **P ≤ 0.01; ***P ≤ 0.001; ****P ≤ 0.0001; *n* = 6.

Targeted metabolomics analysis by UPLC–MS allowed the detection of 44 metabolites, including 5 phenolic and organic acids, 6 amino acids, 9 flavan-3-ols, 7 flavonols, 9 anthocyanins, and 7 stilbenoids (Supplementary Table 2). Unsupervised PCA score plot of the first two components explained 52.9% of the variance and readily discriminated the metabolic profiles of berries from control and Ca-treated vines (Figure 3A). In general, amino acids and anthocyanins di-OH mostly accumulated in control berries, while most phenolic acids were more abundant in berries from Ca-treated vines (Figure 3B). A detailed analysis of berry metabolic profiles showed that all amino acids detected, including L-phenylalanine, were significantly reduced in berries from Ca-treated vines, decreasing by up to 40% in comparison to the control fruits (Figure 4). In contrast, phenolic acids increased by up to 1.9-fold, among which coutaric, caftaric, and fertaric acids. The levels of citric and gallic acids were not affected by Ca treatment. Regarding stilbenoids, a significant increase of 1.8-fold was observed in *E*-piceid levels upon Ca treatment, together with a 6.5-fold increase in *E*-ω-viniferin content. For the remaining stilbenoids, which included *E*-resveratrol, *E*-ε-viniferins, and *E*-δ-viniferins, a tendential but not significant decrease was observed. Regarding flavan-3-ols, only the content in epicatechin was significantly affected by Ca treatment, for which a reduction of 20% was observed. Thus, the apparent increase in procyanidin levels observed in the loading plot was not statistically significant (Figure 3B). The accumulation of specific flavonols, namely kaempferol-3-*O*-rutinoside, quercetin-3-*O*-glucuronide, and myricetin-hexoside 2, was favored by the Ca treatment and their content increased by up to 2.8-fold in comparison to the control fruits. The corresponding glucosides were not significantly affected by the Ca treatment, nor myricetin-hexoside 1 (Figure 4). Anthocyanins detected in berries of cv. Loureiro included cyanidin, peonidin, petunidin, delphinidin, and malvidin conjugates, the latter being the most diverse. The Ca treatment specifically reduced cyanidin-3-*O*-glucoside levels by 40% and malvidin-3-*O*-(6-*p*-coumaroyl)-glucoside content by 60%. The effect of Ca on the remaining anthocyanins was not statistically significant.

**FIGURE 5:**
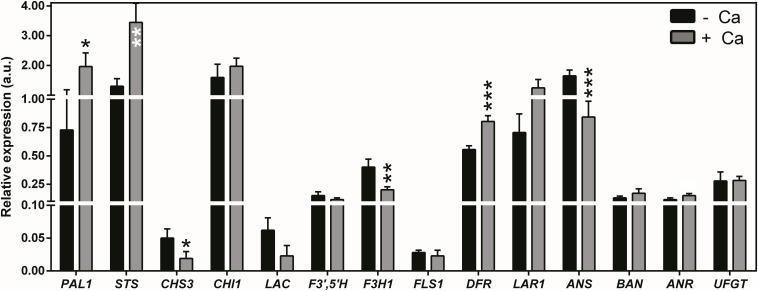
Transcript levels of genes encoding enzymes of secondary metabolism core branches in berries from vines cv. Loureiro treated with Ca (+ Ca) or without treatment (– Ca). Transcript levels are shown for phenylalanine ammonia lyase (*PAL1*), stilbene synthase (*STS*), polyphenol oxidase/laccase (*LAC*), chalcone synthase (*CHS3*), chalcone isomerase (*CHI1*), flavonoid 3′,5′-hydroxylase (*F3′,5′H*), flavanone 3-hydroxylase (*F3H1*), flavonol synthase (*FLS1*), dihydroflavonol 4-reductase (*DFR*), leucoanthocyanidin reductase (*LAR1*), anthocyanidin synthase (*ANS*), anthocyanidin reductases (*BAN* and *ANR*), and anthocyanidin 3-*O*-glucosyltransferase (UDP–glucose:flavonoid-3-*O*-glucosyltransferase, *UFGT*). Expression levels were normalized to the transcript levels of *GAPDH* and (*ACT1*) (housekeeping genes). Results are expressed as mean ± SD and asterisks denote statistical significance as compared to control (– Ca): *P ≤ 0.05; **P ≤ 0.01; and ***P ≤ 0.001; *n* = 3.

**FIGURE 6:**
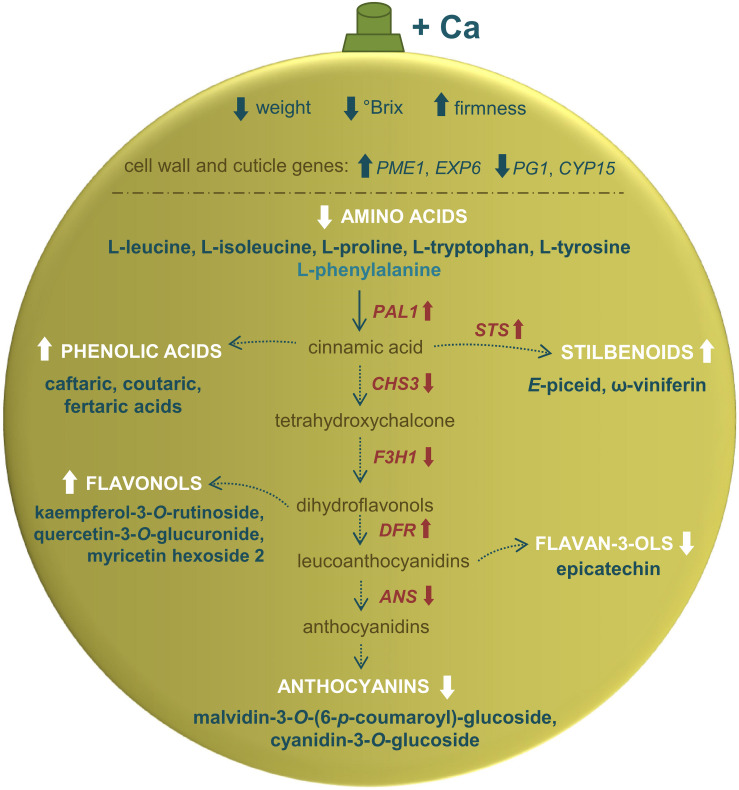
Physiological, metabolic, and transcriptional changes driven by exogenous Ca in grape berries from vines cv. Loureiro. Arrows pointing upward indicate increase in comparison to control berries, while arrows pointing downward indicate decrease. The pathway was based on the information from Kyoto Encyclopedia of Genes and Genomes – KEGG database.

The molecular nature of the metabolic shifts triggered by Ca in berries of cv. Loureiro vines was investigated through the analysis of transcript levels of genes encoding key enzymes of secondary metabolism. The expression of *PAL1*, encoding PAL, was upregulated by 63% upon Ca treatment (Figure 5). Likewise, *STS* encoding stilbene synthases was upregulated by 62%. Coincidentally, the expression of *CHS3* encoding chalcone synthase was 62% lower in berries from Ca-treated vines than in the control berries. The same effect was observed for *F3H1* encoding flavanone 3-hydroxylase, whose transcript levels decreased by 50% upon Ca treatment. Further in the flavonoid pathway, *DFR* encoding dihydroflavonol reductase was upregulated by 30% upon Ca treatment, whereas *ANS* encoding anthocyanidin synthase was downregulated by 49%. Seven other genes of the flavonoid pathway including *UFGT* were not significantly affected by the Ca treatment, nor did *LAC* (laccase) was involved in the *E*-resveratrol oxidation. An overview of the effects of Ca in cv. Loureiro berries is shown in Figure 6.

## Discussion

Results in the present study demonstrated the beneficial effect of Ca sprays over the firmness of berries cv. Loureiro, complementing the few studies conducted in other white cultivars, namely Thompson Seedless, Asgari, and Italia (Amiri et al., 2009; Bonomelli and Ruiz, 2010; Ciccarese et al., 2013), and are in agreement with some previous reports on red cultivars such as Vinhão and Crimson (Alcaraz-López et al., 2005; Martins et al., 2020b). Electron microscopy studies on cv. Vinhão berries showed that increased firmness was accompanied by a reduction in the incidence of microcracks on the fruit surface which became smoother than that of the control fruits (Martins et al., 2020b). The tight regulation of genes involved in the cell wall and cuticle structures by Ca in berries cv. Loureiro likely explained the improved fruit firmness. The observed inhibition of *PG1* and *CYP15* expression was in accordance to previous results in cv. Vinhão berries (Martins et al., 2020b), thus it seems that both white and red varieties share the same targets related to the prevention of fruit softening in response to Ca. PGs degrade pectin molecules in the cell wall and a particularly close correlation between *PG1* levels and grape berry softening has been reported (Deytieux-Belleau et al., 2008). In turn, cuticular CYPs such as *CYP15* are involved in the synthesis of wax triterpenoids, which also determine the fruit quality (Fukushima et al., 2011; Lara et al., 2014). Contrary to previous studies in cv. Vinhão berries (Martins et al., 2020b), *PME1* and *EXP6* were upregulated upon Ca treatment in cv. Loureiro. PME and EXP are involved in various physiological processes underlying both reproductive and vegetative plant development, including seed germination, root tip elongation, and soft fruit ripening (Sampedro and Cosgrove, 2005; Pelloux et al., 2007). PME effects on the latter process arise from its contribution in the degree of demethylated polygalacturonans that are prone to degradation by PGs and the availability of homogalacturonan carboxylic groups for Ca^2+^ binding (Deytieux-Belleau et al., 2008). Accordingly, the induction of PME mRNAs has been associated to the decrease in the degree of methyl-esterification of insoluble pectins during grape berry development (Barnavon et al., 2001).

Results in the present study showed a reduction in the weight and °Brix of mature berries from vines sprayed with Ca, suggesting a delay in fruit maturation, which could be anticipated from the immature appearance of the fruits. This result was not observed in vines of the red cultivar Vinhão subjected to the same Ca application protocol (Martins et al., 2020b). However, decreased °Brix following Ca treatment was reported previously for another white grape cv. Asgari (Amiri et al., 2009). This effect was accompanied by a change in fruit skin color, berries remaining greener, and not attaining the characteristic golden color of ripe fruits (Amiri et al., 2009), much like the observations in the present study with the cv. Loureiro.

In this study, trace amounts of anthocyanins were detected by UPLC–MS in cv. Loureiro berries, as reported in other white cultivars such as Chardonnay, Sauvignon Blanc, Riesling, Pinot Blanc, and Muscat Blanc, also by chromatographic methods (Arapitsas et al., 2015; Niu et al., 2017). In contrast, earlier studies (Boss et al., 1996) reported the absence of these pigments in white cultivars; however, the quantification methods were much less sensitive. Results in the present study and in previous reports suggested that anthocyanin diversity is similar in both white and red grape varieties (Arapitsas et al., 2015; Niu et al., 2017; Martins et al., 2020a). The inhibitory effect of Ca over the anthocyanins malvidin-3-*O*-(6-*p*-coumaroyl)-glucoside and cyanidin-3-*O*-glucoside observed in the present study for fruits cv. Loureiro is in line with previous studies in cv. Vinhão, and is consistent with the downregulation of *ANS* and *UFGT* (Martins et al., 2020a).

The consistent decrease in fruit amino acid levels upon Ca treatment observed in the present study might bring about changes during wine fermentation, as many of these metabolites constitute the yeast assimilable nitrogen fraction of the must (Vilanova et al., 2007). In particular, the decrease in L-phenylalanine levels was tightly linked to the upregulation of *PAL1* encoding the enzyme responsible for its conversion to cinnamic acid, the first catalytic step of plant secondary metabolism (Teixeira et al., 2013). This effect correlated with the increase in phenolic acids, produced in downstream routes initially fed by this substrate. Caftaric acid is known to account for the color of white wines, as it can be hydrolyzed to caffeic acid during the wine-making process, the oxidation of the latter contributing to wine browning (Cilliers and Singleton, 1990). Caftaric acid and other hydroxycinnamates including coutaric acid, also detected in the present study, were shown to be effective markers of wine differentiation, together with resveratrol, piceid, and epicatechin (Andrés-Lacueva et al., 2002; Lampíř, 2013). In the present study, Ca treatment induced *STS* expression and consequently, stilbenoid synthesis, in analogy to previous reports in berries of cv. Vinhão vines located in the same vineyard (Martins et al., 2020a). Although in cv. Vinhão a general increase in most stilbenoids including *E*-resveratrol and *E-ε*-viniferin was reported, in cv. Loureiro a targeted accumulation of *E*-piceid and *E-ω*-viniferin was observed, suggesting a specific action of Ca effect depending on the cultivar. The interaction of Ca with other metabolites differentially present in each cultivar may underlie these effects; accordingly, previous studies showed that the combination of Ca and plant hormones such as jasmonic or abscisic acid greatly determines the redirecting of secondary metabolism toward the synthesis of specific compounds such as different types of viniferins (Martins et al., 2018, 2021b). The targeted action of Ca over specific polyphenols was evident in other metabolic classes, including anthocyanins (discussed above), flavonols, and flavan-3-ols. The large increase of the flavonols kaempferol-3-*O*-rutinoside, quercetin-3-*O*-glucuronide, and myricetin-hexoside 2 observed in cv. Loureiro was not reported previously in cv. Vinhão (Martins et al., 2020a). Flavonols are exclusively found in the grape berry skin and seeds, peaking at veraison stage of fruit development (Teixeira et al., 2013). Thus, the increase in their levels upon Ca treatment supports the delay in fruit maturation in cv. Loureiro, as discussed previously. In parallel, only epicatechin was affected in this cultivar, suggesting a minor influence of Ca over flavan-3-ols contrary to that observed in cv. Vinhão and cv. Gamay Fréaux var. Teinturier cell cultures where a general repression of the flavonoid pathway was reported (Martins et al., 2018, 2020a).

## Conclusion

Results in the present study confirmed the postulated hypothesis, showing that vineyard Ca sprays induce precise metabolic rearrangements in cv. Loureiro berries that result in a substantial delay in fruit maturation. A specific integrated effect of Ca over biochemical and structural properties of cv. Loureiro berries is thus suggested: by inhibiting the action of polygalacturonases responsible for degradation of cell wall pectin and fruit softening, Ca prevents fruit growth and other processes associated with fruit maturation, leading to increased flavonol content and firmness, at the expense of fruit size and °Brix. This effect may be specific for white cultivars, a topic that deserves further investigation. The results may pave the way for the optimization of protocols of Ca treatments in the field aimed to prevent early fruit ripening in specific cultivars from wine regions most affected by climate change, possibly consisting of a good alternative to crop forcing. Additional benefits on the resistance to biotic and abiotic stresses and on shelf-life could also be expected, in accordance to previous studies (Romanazzi et al., 2012; Martins et al., 2021a).

## Data Availability Statement

The original contributions presented in the study are included in the article/Supplementary Material, further inquiries can be directed to the corresponding author.

## Author Contributions

VM and HG conceptualized the work. VM performed field trials, sample processing, firmness measurements, quantification of biochemical parameters, and gene expression analyses. AL and MU performed metabolomic analysis and data treatment. AT performed statistical analysis. HG and AL contributed with resources and funding acquisition. VM, HG, and AL wrote the manuscript. All authors edited and reviewed the manuscript, contributed to the article, and approved the submitted version.

## Conflict of Interest

The authors declare that the research was conducted in the absence of any commercial or financial relationships that could be construed as a potential conflict of interest.

## Publisher’s Note

All claims expressed in this article are solely those of the authors and do not necessarily represent those of their affiliated organizations, or those of the publisher, the editors and the reviewers. Any product that may be evaluated in this article, or claim that may be made by its manufacturer, is not guaranteed or endorsed by the publisher.

## Supplementary Material

Supplementary Figure 1 Edaphoclimatic conditions of the vineyard located in the DOC region ‘Vinhos Verdes’ where field trials were conducted.

Supplementary Table 1 Accession numbers and specific primers forward (F) and reverse (R) of sequences used in quantitative real-time PCR studies.

Supplementary Table 2 Polyphenolic compounds quantified in mature grape berries from cv. Loureiro vines treated with Ca (+ Ca) or without treatment (− Ca).
