# Supplementary material for: Exogenous Calcium Delays Grape Berry Maturation in the White cv. Loureiro While Increasing Fruit Firmness and Flavonol Content
Source: Front Plant Sci. 2021 Aug 27;12:742887. doi: 10.3389/fpls.2021.742887 (PMC8430324; doi:10.3389/fpls.2021.742887)
Supplement: Supplementary Figure 1 — Edaphoclimatic conditions of the vineyard located in the DOC region ‘Vinhos Verdes’ where field trials were conducted. [file Data_Sheet_1.PDF]

Exogenous calcium delays grape berry maturation in the white cv. Loureiro while increasing fruit firmness and flavonol content

Viviana Martins[1,2*], Marianne Unlubayir[3], António Teixeira[1], Arnaud Lanoue[3], Hernâni Gerós[1,2,4]

[1]Centre of Molecular and Environmental Biology, Department of Biology, University of Minho, Campus de Gualtar, 4710-057 Braga, Portugal
[2]Centre for the Research and Technology of Agro-Environmental and Biological Sciences, University of Trás-os-Montes and Alto Douro, 5001-801 Vila Real, Portugal
[3]Université de Tours, EA 2106 «Biomolécules et Biotechnologie Végétales», UFR des Sciences Pharmaceutiques, 31 Av. Monge, F37200 Tours, France
[4]Centre of Biological Engineering (CEB), Department of Biological Engineering, University of Minho, Campus de Gualtar, 4710-057 Braga, Portugal

***Correspondence:**
Viviana Martins, Centre of Molecular and Environmental Biology, Department of Biology, University of Minho, Campus de Gualtar, 4710-057 Braga, Portugal, 00 351 253 604048, vmartins@bio.uminho.pt

Supplementary Material

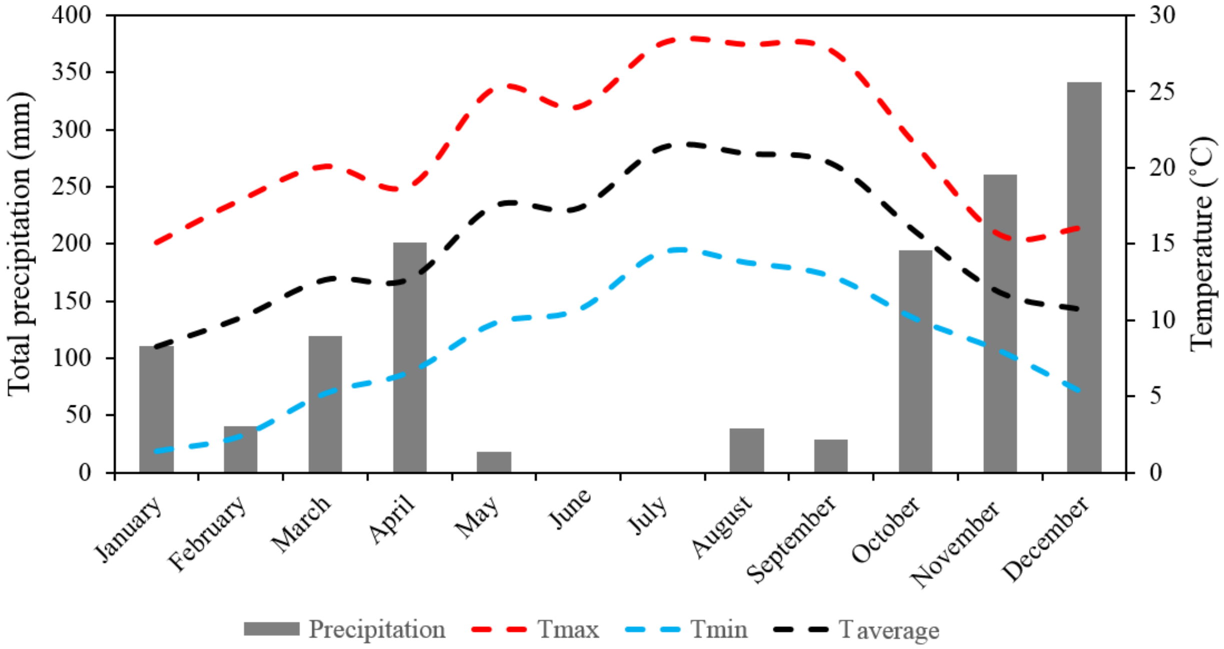

Figure S1. Edaphoclimatic conditions of the vineyard located in the DOC region 'Vinhos Verdes', where the field trials were conducted. Data was retrieved from the Portuguese IPMA (*Instituto Português do Mar e da Atmosfera*) relative to the year of 2019, at https://www.ipma.pt/pt/publicacoes/.

Table S1. Accession numbers and specific primers forward (F) and reverse (R) of sequences used in qPCR studies.

NCBI/Genbank Accession Number	Name	Sequence (5' – 3')	References
XM_002281763.4	*PAL1*	**F** CCGAACCGAATCAAGGACTG<br>**R** GTTCCAGCCACTGAGACAAT	Boubakri et al. (2013)
NM_001281117.1	*STS**	**F** CGAAGCAACTAGGCATGTGT<br>**R** CTCCCCAATCCAATCCTTCA	Boubakri et al. (2013)
XM_002263983.3	*CHS3*	**F** TCGCATCACAAATAGCGAAC<br>**R** CAGGGAAGCTGCCATGTATT	Ageorges et al. (2006)
GSVIVT00012329001[#]	*LAC*	**F** AGAAGTATCCAGCACGTTTACGAGT<br>**R** CCCCTGTTCTGGTGTCAATAAAGTA	Tavares et al. (2013)
X75963	*CHI1*	**F** CAGGCAACTCCATTCTTTTC<br>**R** TTCTCTATCACTGCATTCCC	Jeong et al. (2004)
GSVIVT00022298001[#]	*F3'5'H*	**F** CATCAAGCGTAATCGAGTGGTCTCT<br>**R** CCTTGCATATGGCTTGTAGGTATGG	Tavares et al. (2013)
X75965	*F3H1*	**F** CCAATCATAGCAGACTGTCC<br>**R** TCAGAGGATACACGGTTGCC	Jeong et al. (2004)
XM_002285802.3	*FLS1*	**F** CAGGGCTTGCAGGTTTTTAG<br>**R** GGGTCTTCTCCTTGTTCACG	Downey et al. (2003)
X75964	*DFR*	**F** GGCTTTCTAGCGAGAGCGTA<br>**R** ACTCTCATTTCCGGCACATT	Bogs et al. (2006)
AJ865336	*LAR1*	**F** CAGGAGGCTATGGAGAAGATAC<br>**R** ACGCTTCTCTCTGTACATGTTG	Bogs et al. (2015)
GSVIVT00019892001[#]	*ANS*	**F** CAACAATGCTAGTGGACAGCTTGAG<br>**R** TGGAACGTAGTCGCTTGGTGTCTTA	Tavares et al. (2013)
BN000166	*BAN*	**F** GCAGGTTGCGACTTTGTCTTCC<br>**R** TGGCTTGATCATGTCATTCTCTGG	Martins et al. (2021)
NM_001280956.1	*ANR*	**F** AGCAGGTTGCGACTTTGTCT<br>**R** ACCAGACCTGTCCCATCAAG	Bogs et al. (2015)
XM_002276999.4	*UFGT*	**F** TGCAGGGCCTAACTCACTCT<br>**R** GCAGTCGCCTTAGGTAGCAC	Conde et al. (2016)
AF159122	*PME1*	**F** GGATACCAGGACACACTGT<br>**R** CTTCCATGGCCTACCGAGGTA	Martins et al. (2018)
AY043233	*PG1*	**F** GAATCAAGACATGGCAGG<br>**R** TAATCCTAGCTTCCATGCA	Martins et al. (2018)
XM_002283494	*EXP6*	**F** AGCGTAAAAGGGTCGAAGACAG<br>**R** CGATGTTCCAGGATGTTGATGT	Fernandes et al. (2016)
XM_002278961	*CesA3*	**F** CGAGGGGAGGATACAAACTACG<br>**R** ATAGAAAGACGCTCGGGTGAAG	Fernandes et al. (2016)
XM_010649426.1	*CER9*	**F** TGGAAGCATTCATGCATCAGG<br>**R** AGCACAAAGCCGTACTCTGA	Martins et al. (2020)

NM_001281186.1	*CYP15*	**F** GATGCCACATGAATGAAATGGAAA<br>**R** TGATAAGGAATGTAATGGCAGCA	Martins et al. (2020)
XM_002263109	*GAPDH*	**F** TTCCGTGTTCCTACTGTTG<br>**R** CCTCTGACTCCTCCTTGAT	Gainza-Cortés et al. (2012)
XM_002282480	*ACT1*	**F** CTTGCATCCCTCAGCACCTT<br>**R** TCCTGTGGACAATGGATGGA	Le Henanff et al. (2009)

[#]NCBI/Genbank accession unavailable

* the primers for *STS* are not specific for this accession and amplify several STS family genes, providing a broader overview of the changes in this metabolic pathway.

References

Ageorges, A., Fernandez, L., Vialet, S., Merdinoglu, D., Terrier, N., & Romieu, C. (2006). Four specific isogenes of the anthocyanin metabolic pathway are systematically co-expressed with the red colour of grape berries. Plant Science, 170(2), 372-383.

Bogs, J., Downey, M. O., Harvey, J. S., Ashton, A. R., Tanner, G. J., & Robinson, S. P. (2005). Proanthocyanidin synthesis and expression of genes encoding leucoanthocyanidin reductase and anthocyanidin reductase in developing grape berries and grapevine leaves. Plant Physiology, 139(2), 652-663.

Boubakri, H., Poutaraud, A., Wahab, M. A., Clayeux, C., Baltenweck-Guyot, R., Steyer, D., et al. (2013). Thiamine modulates metabolism of the phenylpropanoid pathway leading to enhanced resistance to *Plasmopara viticola* in grapevine. BMC Plant Biology, 13(1), 31.

Conde, A., Pimentel, D., Neves, A., Dinis, L. T., Bernardo, S., Correia, C. M., et al. (2016). Kaolin foliar application has a stimulatory effect on phenylpropanoid and flavonoid pathways in grape berries. Frontiers in Plant Science, 7, 1150.

Downey, M. O., Harvey, J. S., & Robinson, S. P. (2003). Synthesis of flavonols and expression of flavonol synthase genes in the developing grape berries of Shiraz and Chardonnay (*Vitis vinifera* L.). Australian Journal of Grape and Wine Research, 9(2), 110-121.

Fernandes, J. C., Goulao, L. F., & Amâncio, S. (2016). Regulation of cell wall remodeling in grapevine (*Vitis vinifera* L.) callus under individual mineral stress deficiency. Journal of Plant Physiology, 190, 95-105.

Gainza-Cortés, F., Pérez-Dïaz, R., Pérez-Castro, R., Tapia, J., Casaretto, J. A., González, S., Peña-Cortés, H., Ruiz-Lara, S., & González, E. (2012). Characterization of a putative grapevine Zn transporter, VvZIP3, suggests its involvement in early reproductive development in *Vitis vinifera* L. BMC Plant Biology, 12(1), 111.

Jeong, S. T., Goto-Yamamoto, N., Kobayashi, S., & Esaka, M. J. P. S. (2004). Effects of plant hormones and shading on the accumulation of anthocyanins and the expression of anthocyanin biosynthetic genes in grape berry skins. Plant Science, 167(2), 247-252.

Le Henanff, G., Heitz, T., Mestre, P., Mutterer, J., Walter, B., & Chong, J. (2009). Characterization of *Vitis vinifera* NPR1 homologs involved in the regulation of pathogenesis-related gene expression. BMC Plant Biology, 9(1), 54.

Martins, V., Garcia, A., Costa, C., Sottomayor, M., & Gerós, H. (2018). Calcium-and hormone-driven regulation of secondary metabolism and cell wall enzymes in grape berry cells. Journal of Plant Physiology, 231, 57-67.

Martins, V., Garcia, A., Alhinho, A.T., Costa, P., Lanceros-Méndez, S., Costa, M.M.R., & Gerós, H. (2020). Vineyard calcium sprays induce changes in grape berry skin, firmness, cell wall composition and expression of cell wall-related genes. Plant Physiology and Biochemistry, 150, 49-55.

Martins, V., Unlubayir, M., Teixeira, A., Gerós, H., & Lanoue, A. (2021). Calcium and methyl jasmonate cross-talk in the secondary metabolism of grape cells. Plant Physiology and Biochemistry, 165, 228-238.

Tavares, S., Vesentini, D., Fernandes, J. C., Ferreira, R. B., Laureano, O., Ricardo-Da-Silva, J. M., & Amâncio, S. (2013). *Vitis vinifera* secondary metabolism as affected by sulfate depletion: diagnosis through phenylpropanoid pathway genes and metabolites. Plant Physiology and Biochemistry, 66, 118-126.

Table S2. Polyphenolic compounds quantified in mature grape berries from cv. Loureiro vines treated with Ca (+ Ca) or without treatment (- Ca). Results show mean ± SD of relative values (AU mg^{-1} dry weight: arbitrary units of metabolite per mg of sample dry weight) obtained for six biological replicates per treatment.

Class	Metabolite	- Ca			+ Ca		
		mean		SD	mean	±	SD
Amino acids	L-proline	43370.14	±	5585.33	31885.01	±	3456.96
	L-leucine	86220.15	±	6701.23	66847.89	±	3498.43
	L-isoleucine	138830.55	±	14215.12	105923.46	±	5092.81
	L-phenylalanine	130541.19	±	8941.96	78607.73	±	5315.81
	L-tyrosine	10357.94	±	1213.06	6651.34	±	934.41
	L-tryptophan	184745.25	±	17122.81	154752.68	±	10702.88
Phenolic acids	gallic acid	283.18	±	34.12	246.68	±	22.16
	citric acid	4363.14	±	632.16	4419.13	±	299.17
	coutaric acid	4432.01	±	292.62	7594.90	±	945.43
	caftaric acid	13244.91	±	1692.09	18968.81	±	1306.41
	fertaric acid	722.17	±	92.29	1366.18	±	120.90
Stilbenoids DP1	*E* -resveratrol	632.05	±	606.37	75.99	±	54.15
	piceatannol	64.68	±	61.53	15.40	±	10.35
	E -piceid	286.12	±	54.08	507.40	±	130.88
Stilbenoids DP2	pallidol	161.81	±	94.24	83.84	±	22.76
	E-ε-viniferin	268.16	±	161.02	158.89	±	43.78
	E-ω-viniferin	6.90	±	2.34	44.56	±	20.23
	E-δ-viniferin	24.44	±	7.76	17.60	±	5.46
Flavan-3-ols	catechin	16022.78	±	2060.49	15434.73	±	2438.19
	epicatechin	10740.17	±	1152.37	8689.81	±	945.14
	procyanidinB1	1139.38	±	222.92	1404.65	±	218.20
	procyandinB2	562.02	±	369.69	728.53	±	130.14
	procyanidinB3	1504.11	±	200.45	1275.91	±	260.14
	procyanidinB4	1825.66	±	436.95	1573.65	±	293.86
	procyanidin gallate	2155.19	±	464.63	2399.51	±	581.10
	procyanidin trimer1	714.42	±	122.32	865.07	±	118.23
	procyanidin trimer 2	2283.74	±	291.24	2590.37	±	655.24
Flavonols	quercetin 3-*O* - glucoside	877.99	±	39.98	730.96	±	159.09
	quercetin-3-*O* -glucuronide	2504.42	±	451.86	5630.50	±	614.49
	myricetin-hexoside 1	13.33	±	4.93	10.81	±	3.78
	myricetin-hexoside 2	28.94	±	7.39	65.66	±	19.19
	quercetin-conjungate	5.89	±	2.80	3.84	±	2.35
	kaempferol glucoside	7.03	±	4.95	4.59	±	4.49
	kaempferol-3-*O* -rutinoside	21.52	±	7.65	59.94	±	26.66
Anthocyanins di-OH	cyanidin-3-*O*- glucoside	359.62	±	65.61	216.81	±	19.24
	peonidin-3-*O* -glucoside	267.75	±	66.71	241.39	±	40.41
	cyanidin-3-*O* -(6-*O* -acetyl)-glucoside	232.38	±	46.28	230.08	±	31.44
Anthocyanins tri-OH	delphinidin-3-*O* -glucoside	92.26	±	30.18	122.72	±	92.55
	malvidin-3-*O* -glucoside	731.95	±	259.52	575.32	±	205.65
	malvidin-3-*O* -(6-*O*- acetyl)-glucoside	154.87	±	62.17	143.67	±	49.91
	petunidin-3-*O* -(6-*p* -coumaroyl)-glucoside	89.40	±	12.44	119.40	±	64.68
	malvidin-3-*O* -(6-*p* -coumaroyl)-glucoside	242.78	±	119.39	99.14	±	65.19
	malvidin-3,5-*O*- diglucoside	39.84	±	11.25	36.27	±	19.13
